# The Impact of a Single Stretching Session on Running Performance and Running Economy: A Scoping Review

**DOI:** 10.3389/fphys.2020.630282

**Published:** 2021-01-20

**Authors:** Andreas Konrad, Richard Močnik, Masatoshi Nakamura, Karl Sudi, Markus Tilp

**Affiliations:** ^1^Institute of Human Movement Science, Sport and Health, University of Graz, Graz, Austria; ^2^Institute for Human Movement and Medical Sciences, Niigata University of Health and Welfare, Niigata, Japan

**Keywords:** running economy, running performance, oxygen uptake, stretching, mobility

## Abstract

One determining factor for running performance is running economy (RE), which can be quantified as the steady-state oxygen consumption at a given running speed. Stretching is frequently applied in sports practice and has been widely investigated in recent years. However, the effect of stretching on RE and performance is not clear. Thus, the purpose of this scoping review is to investigate the effects of a single bout of stretching on RE and running performance in athletes (e.g., recreational and elites) and non-athletes. The online search was performed in PubMed, Scopus, and Web of Science databases. Only studies that explored the acute effects of stretching on RE (or similar variables) and/or running performance variables with healthy and adult participants, independent of activity level, were included in this review. Eleven studies met the inclusion criteria with a total of 44 parameters (14 performance-related/30 metabolic parameters) and 111 participants. Regardless of the stretching technique, there was an improvement both in performance variables (21.4%) and metabolic variables (13.3%) following an acute bout of stretching. However, detrimental effects in performance variables (28.5%) and metabolic variables (6.6%) were also reported, though the results were influenced by the stretching duration and technique. Although it was observed that a single static stretching exercise with a duration of up to 90 s per muscle group can lead to small improvements in RE (1.0%; 95% CI: −1.04 to 2.22), negative effects were reported in running performance (−1.4%; 95% CI: −3.07 to −0.17). It was also observed that a single bout of dynamic stretching only resulted in a negligible change in RE −0.79% (95% CI: −0.95 to 4.18) but a large increase in running performance (9.8%; 95% CI: −3.28 to 16.78), with an overall stretch duration (including all muscles) between 217 and 900 s. Therefore, if stretching is applied without additional warm-up, the results suggest applying dynamic stretching (for a short overall stretching duration of ≤220 s) rather than static stretching if the goal is to increase running performance. In general, only short static stretching durations of ≤60 s per muscle–tendon unit are advisable. One study reported that less flexible runners have greater benefits from stretching than athletes with normal flexibility. In addition, it can be suggested that less flexible runners should aim for an optimum amount of flexibility, which would likely result in a more economical run.

## Introduction

Running is one of the most popular sports worldwide, alongside soccer, walking, and athletics (Hulteen et al., 2017). One determining factor for running performance is running economy (RE), which can be quantified as the steady-state oxygen consumption at a given running speed (Barnes and Kilding, 2015). RE can be increased by long-term interventions such as resistance training (e.g., plyometric training, strength training) or even bouts of high-intensity runs performed on a flat surface or uphill (Barnes and Kilding, 2015). Running is characterized by a complex interaction of several muscle–tendon units, which adapt their mechano-morphological structure due to the chronic stress induced by running. One of the most important properties of the muscle and tendon/aponeurosis structure is their stiffness (Saunders et al., 2004). However, the influence of tissue stiffness on running performance is complex. On the one hand, there is evidence that a more compliant quadriceps tendon and aponeurosis is associated with better RE in endurance athletes (Arampatzis et al., 2006). On the other hand, stiffer tendons of the plantar flexors (Arampatzis et al., 2006) and stiffer muscle–tendon units of the hamstrings (Jones, 2002) are also associated with better RE. Changes in tissue stiffness, which would likely affect RE (positively and negatively), can be achieved in both long-term and acute interventions, such as using stretching exercises (Kay et al., 2015; Konrad et al., 2015, 2017).

With regard to long-term interventions, it is recommended that endurance athletes perform jumping training (e.g., plyometrics) and heavy weight training to improve RE and hence running performance. Although it is recommended that such training be performed for at least 6–8 weeks, a longer time period (>8 weeks) results in a greater change in RE (Denadai et al., 2017). Furthermore, the suggested types of resistance training exercises that are frequently performed for a 12-week period can affect both the mechanical properties of the muscle–tendon unit (e.g., stiffness) and the function of the muscle–tendon unit (Kubo et al., 2007). Stretching interventions for several weeks can increase the range of motion (RoM) of a joint (Mahieu et al., 2009; Konrad and Tilp, 2014b). This change in RoM can be attributed not only to decreases in tendon (Konrad et al., 2015) or muscle (Nakamura et al., 2017) stiffness but also to an altered perception of stretch or pain (Konrad and Tilp, 2014a). Barnes and Kilding (2015) assumed that decreases in soft tissue stiffness following long-term stretching interventions might lead to a detrimental effect in running (Barnes and Kilding, 2015). However, Nelson et al. (2001) reported that a stretching intervention for 10 weeks resulted in no changes in RE. Besides long-term interventions, endurance athletes commonly perform acute exercises during warm-up in the belief that this positively affects running performance and/or RE. Single muscle contractions (Kubo et al., 2001), single stretching sessions (Konrad et al., 2017), and even massages (Konrad et al., 2020) can change the mechanical properties of the muscle–tendon unit (e.g., tissue stiffness) and the function of the muscle–tendon unit (e.g., joint flexibility, strength) acutely. Single stretching sessions, in particular, are a frequently used modality among endurance athletes as part of a warm-up routine (Bishop, 2003). However, the different stretching techniques and the diversity of the duration are subjects of some controversy in the literature. With regard to stretching duration, a review by Behm et al. (2016) reported a greater loss in performance (strength tasks) with static stretching of ≥60 s (−4.6%) compared to static stretching of <60 s (−1.1%). With regard to the different stretching techniques which can be applied, Behm et al. (2016) reported mean performance impairments (in strength tasks) of 3.7 and 4.4% immediately after static stretching and proprioceptive neuromuscular facilitation (PNF) stretching, respectively, but an increase in performance of 1.3% after dynamic stretching. However, to date, no review has reported the effects of a single bout of stretching, with its different variations in terms of duration and technique, for running variables such as running performance or RE.

Therefore, the purpose of this scoping review is to summarize the existing evidence about the effects of a single bout of stretching on running performance parameters (e.g., running distance, stride length) and/or RE (e.g., O_2_ uptake) in athletes (e.g., recreational and elites) and non-athletes.

## Materials and Methods

This review is based on the suggestions from Munn et al. (2018) for scoping reviews. Thus, the purposes of this review were to identify the available evidence and to identify knowledge gaps. This review considers scientific papers which investigated the effect of all types of stretching on RE and/or running performance. The electronic literature search was performed in three different databases (PubMed, Scopus, and Web of Science) and was conducted on the 18th of August 2020. Two researchers performed this search by using the following search terms: [(“running economy” OR “running performance” OR VO_2_^∗^ OR “oxygen uptake” OR “energy cost^∗^”) AND stretch^∗^]. The search only considered studies which were written in the English language. This process resulted in a total number of 1043 studies being identified.

After removing duplicates (477), the remaining studies were screened by title (or, if necessary, by abstract) to identify the studies to be included in this review. Overall, 566 studies were screened, and 11 meet the inclusion criteria and, hence, were included in this review. Only studies that explored the acute effects of stretching on RE (or similar variables) and/or running performance variables with healthy and adult participants, independent of activity level, were included in this review.

**FIGURE 1:**
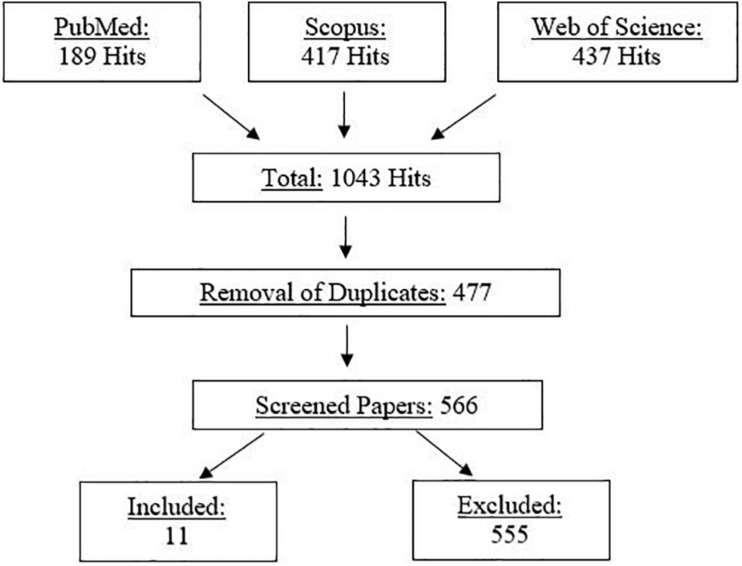
Flowchart of the systematic screening process (PRISMA).

A detailed illustration of the search process is provided in Figure 1.

In order to analyze the effect of the stretching duration of the static stretching technique, we classified the stretching duration into 20–90 and >120 s. Moreover, the mean percentage changes and the 95% confidence intervals (CI) of the included studies are presented in the results.

## Results

Eleven studies of the acute effects of stretching on RE and running performance were included in this review. These studies included a total of 111 subjects (male, 99; female, 12), with an average age of 25.3 (±5.2) years. Table 1 shows detailed information about the population, the stretching exercises applied, and the outcomes of all the measured parameters in the included studies. Out of the 11 studies, we identified 14 running-related performance parameters (e.g., running time at a given distance, stride length) and 30 metabolic parameters (e.g., RE, O_2_ uptake, heart rate) (see Table 2). Three of the performance parameters (21.4%) showed an improvement, seven showed no change (50.0%), and four showed an impairment (28.6%) following the single stretching sessions (for more details, see Table 2). With regard to the metabolic parameters, four parameters (13.3%) showed an improvement, 24 (80.0%) showed no change, and two (6.7%) parameters showed an impairment following a single bout of stretching (for more details, see Table 2). Overall, there was an improvement in 15.9% and an impairment in 13.6% of the investigated parameters.

**TABLE 1:** Summary of the results and summary of the participants and intervention characteristics of the studies which investigated the acute effects of stretching on running economy and running performance.

**Study**	**Subjects**	**Stretching intervention**		**Main outcome in % change**
		**Muscle (group)**	**Type/duration**	
Allison et al., 2008	Ten male runners (Ø VO_2_ max. 60.1 ± 7.3 ml/kg/min) Age: 25 ± 5	Lower body	Eight static stretches of 4 × 40 s (unilateral)	↑ RoM (sit and reach) (nr% pre to post)
				↓ CMJ height (5.5% pre to post)
				↓ Isometric strength (5.6% pre to post)
				↔ Changes in oxygen uptake (0% pre to post)
				↔ Changes in minute ventilation (+2.26% pre to post)
				↔ Changes in energy expenditure (+0.65% pre to post)
				↑ RoM (sit and reach) (nr; compared with the control condition)
				↓ Drop jump height (nr; compared with the control condition)
Damasceno et al., 2014	Eleven male long distance runners (Ø VO_2_ max. 51.0 ± 3.0 ml/kg/min) Age: 35.7 ± 6.1	Lower body	Seven static stretches of 3 × 30 s each	Constant speed test variables (at 12 km/h; all compared to the control condition)
				↔ Changes in running economy (−2.22%)
				↔ Changes in caloric unit cost (−3.00%)
				↑ Stride time (1.83%)
				↔ Changes in contact time (−2.40%)
				↔ Changes in flight time (+1.99%)
				↑ iEMG of biceps femoris (22.34%)
				↔ Changes in iEMG of gastrocnemius med. (+19.20%)
				↔ Changes in iEMG of vastus med. (6.25%)
				Time/trial test variables (for 3 km)
				↔ Changes in overall running time (+1.01%)
				↑ RoM (hip flexion): static (28.92%), PNF (15.58%) (pre to post);
				↑ RoM (hip extension): static (36.36%), PNF (69.23%) (pre to post)
Godges et al., 1989	Seven males	Hip extensors	–>Static stretching group: 10 min	Oxygen consumption following static stretching:
	Age: 20		–>PNF stretching group: like static, but including soft tissue mobilization	↓ Oxygen consumption at 40% of VO_2_ max (6.64%) (pre to post)
				↓ Oxygen consumption at 60% of VO_2_ max (4.16%) (pre to post)
				↓ Oxygen consumption at 80% of VO_2_ max (3.83%) (pre to post)
				Oxygen consumption following PNF stretching:
				↔ Changes in oxygen consumption at 40% of VO_2_ max
				(−3.14%) (pre to post)
				↓ Oxygen consumption at 60% of VO_2_ max (3.75%) (pre to post);
				↔ Changes in oxygen consumption at 80% of VO_2_ max
				(−2.34%) (pre to post)
Hayes and Walker, 2007	Seven male middle and long distance runners (Ø VO_2_ max. 66.8 ± 7.0 ml/kg/min) Age: 32.5 ± 7.7	Lower body	five exercises	In all three techniques
			@ 2 × 30 s for:	↑RoM (sit and reach): static (nr), progressive static (nr), dynamic (nr) (pre to post)
			>Static stretching	↔ Changes in running economy: static (1.04%)
			>Progressive static stretching	Progressive static (1.83%), dynamic (0.86%) (compared with the control condition)
			>Dynamic stretching	↔ Changes in steady-state oxygen uptake
				Static: nr, progressive static; nr, dynamic: nr (compared with the control condition) ↑ RoM (sit and reach) (17.22% pre to post)
Lowery et al., 2014	Ten male distance runners (Ø VO_2_ max 64.9 ± 6.5 ml/kg/min) Age: 24 ± 5	Lower body	Six static stretches of 3 × 30 s each	↓ One-mile uphill run time (3.07% compared with the control condition)
				↑ Ground contact time (12.58% pre to post)
				↑ Muscle activation (15.71% pre to post)
				↑ RoM (sit and reach) (pre to post 11.07%)
Mojock et al., 2011	Twelve female long distance runners (Ø VO_2_ max 48.4 ±5.1 ml/kg/min Age: 30 ± 9	Lower body	Five static stretches of 2 × 60 s each	Preload run variables (at 65% VO_2_ max—compared to the control condition)
				↔ Changes in average heart rate (+1.88%)
				↔ Changes in rate of perceived exertion (0%)
				↔ Changes in energy expenditure (0%)
				↔ Changes in 65% VO_2_ (+0.29%)
				Performance run variables (compared to the control condition)
				↔ Changes in average heart rate (+1.13%)
				↔ Changes in heart rate max (+0.53%)
				↔ Changes in rate of perceived exertion average (0%)
				↔ Changes in rate of perceived exertion max (0%)
				↔ Changes in running speed (0%)
				↔ Changes in covered distance (−0.18%)
Takizawa et al., 2015	Seven male middle or long distance runners (Ø VO_2_ max. 72.3 ±3.7 ml/kg/min) Age: 21.3 ± 2.1	Lower body	Five static stretches of 1 × 20 s each	↔ Changes at time to exhaustion at 90% of VO_2_ max
				(−0.17% compared with the control condition)
				↔ Changes in oxygen uptake (nr% compared with the control condition)
				↔ Changes in vastus lateralis temperature after stretching (−1.09% compared with the control condition)
				↔ Changes in blood lactate accumulation after stretching (+11.00% compared with the control condition)
				↔ Changes in blood lactate accumulation after performance run (+5.21% compared with the control condition)
Wilson et al., 2010	Ten male distance runners (Ø VO_2_ max 63.8 ± 2.8 ml/kg/min) Age: 25 ± 7	Lower body	Five static stretches of 4 × 30 s each	↑ Performance in a 30-min run (3.44% compared with the control condition) ↑ Energy expenditure during 30-min performance run (4.71% compared with the control condition)
Yamaguchi et al., 2015	Seven male middle or long distance runners (Ø VO_2_ max. 72.3 ± 3.7 ml/kg/min) Age: 21.3 ± 2.1	Lower body	Five dynamic stretches with 10 reps as fast as possible (total = 217 ± 17 s)	↑ Time to exhaustion (15.43% compared with the control condition)
				↑ Total running distance (15.91% compared with the control condition) ↔ Changes in VO_2_ (−0.95% compared with the control condition) ↔ Changes in lactate (+ 8.39% compared with the control condition)
				↔ Changes in heart rate (+0.11% compared with the control condition)
Yamaguchi et al., 2020	Sixteen male long distance runners (Ø VO_2_ max. 71.9 ± 3.6 ml/kg/min) Age: 20.9 ± 2.1	Lower body	Five dynamic stretches with 10 reps as fast as possible (total = 220 ± 9 s)	↑ Time to exhaustion (16.78% compared with the running warm-up)
				↔ Differences in VO_2_ uptake between dynamic stretching group and running warm-up group during assessment of running performance
Zourdos et al., 2012	Fourteen male trained runners (VO_2_ max 63.1 ± 8.3 ml/kg/min) Age: 23.0 ± 4.3	Lower body	Ten dynamic stretches with 2 × 4 reps (total = 900 s)	↑ RoM (sit and reach) (16.41% pre to post)
				↑ Resting VO_2_ (35.48% pre to post)
				↑ Energy cost during 30-min preload run (4.18% compared with the control condition)
				↔ Changes in the distance run (−3.28% compared with the control condition)

*RoM, range of motion; CMJ, countermovement jump; ↑, significant increase; ↓, significant decrease; ↔, no significant change; nr, not reported.*

**TABLE 2:** Direction of change in running performance or metabolic parameters of the included studies.

**Study**	**Parameter**	**Outcome**
**Performance parameters**		
Yamaguchi et al., 2015	Time to exhaustion	Improvement
	Total running distance	Improvement
Yamaguchi et al., 2020	Time to exhaustion	Improvement
Damasceno et al., 2014	Stride time	Impairment
Lowery et al., 2014	One mile uphill run time	Impairment
	Ground contact time	Impairment
Wilson et al., 2010	30-Min running performance	Impairment
Damasceno et al., 2014	Contact time	No change
	Flight time	No change
	3k time trial	No change
Mojock et al., 2011	Running speed	No change
	Distance covered	No change
Takizawa et al., 2015	Time to exhaustion	No change
Zourdos et al., 2012	Total running distance	No change
**Metabolic parameters**		
Godges et al., 1989	Oxygen uptake @40% VO_2_ maximum static stretching	Improvement
	Oxygen uptake @60% VO_2_ maximum static stretching	Improvement
	Oxygen uptake @80% VO_2_ maximum static stretching	Improvement
	Oxygen uptake @60% VO_2_ maximum proprioceptive neuromuscular facilitation (PNF) stretching	Improvement
Wilson et al., 2010	Energy expenditure	Impairment
Zourdos et al., 2012	Energy cost during 30-min run	Impairment
Allison et al., 2008	Oxygen uptake	No change
	Minute ventilation	No change
	Energy expenditure	No change
Damasceno et al., 2014	Running economy	No change
	Caloric unic cost	No change
Godges et al., 1989	Oxygen uptake @40% VO_2_ maximum PNF stretching	No change
	Oxygen uptake @80% VO_2_ maximum PNF stretching	No change
Hayes and Walker, 2007	Running economy static stretching	No change
	Oxygen uptake static stretching	No change
	Running economy progressive static stretching	No change
	Oxygen uptake progressive static stretching	No change
	Running economy dynamic stretching	No change
	Oxygen uptake progressive dynamic stretching	No change
Mojock et al., 2011	Average heart rate @65% of VO_2_ maximum	No change
	Energy expenditure @65% VO_2_ maximum	No change
	65% VO_2_ maximum	No change
	Average heart rate at 30 min all-out test	No change
	Heart rate maximum at 30 min all-out test	No change
Takizawa et al., 2015	Oxygen uptake	No change
	Blood lactate accumulation	No change
Yamaguchi et al., 2015	Oxygen uptake	No change
	Blood lactate concentration	No change
	Heart rate	No change
Yamaguchi et al., 2020	Oxygen uptake	No change

For the studies which investigated RE exclusively (or equivalent), a single bout of stretching led to an average improvement of 1.3% (*n* = 8). With regard to running performance parameters (covered distance or time to exhaustion), a single bout of stretching led to an average improvement of 2.7% in nine studies.

However, these pooled results do not consider the stretching duration or the stretching technique used.

### Stretching Duration

The duration of the stretching of a single isolated muscle group can only be quantified for static stretching because dynamic stretching is performed with complex movement (whole-body movement). Thus, it is impossible to detect the exact duration of the stretches performed on isolated muscle groups during dynamic stretching. The static stretching interventions lasted from 20 to 600 s (total stretching duration). In order to analyze the effect of the stretching duration, we classified the static stretching duration into 20–90 and ≥120 s. The studies including a stretching regimen of 20–90 s showed a small overall improvement in RE (1.0%; 95% CI: −1.04 to 2.22; *n* = 3). The studies including stretching durations of ≥120 s showed, on average, an impairment of −0.03% (95% CI: −3.53 to 3.59; *n* = 4). With regard to running performance, static stretching durations of up to 90 s (*n* = 3) led to an average decrease of −1.4% (95% CI: −3.07 to −0.17), while longer durations (≥120 s; *n* = 2) led to an average decrease of −1.8% (95% CI: −3.44 to −0.18).

### Stretching Method

Static stretching led to an average improvement in RE (or equivalent) of 0.4% (95% CI: −1.91 to 2.63), based on data from six studies. Moreover, an average decrease in running performance of −1.6% (95% CI: −2.81 to −0.34; *n* = 5) was found following a single bout of static stretching. For a single bout of dynamic stretching, RE decreased by −0.79% (95% CI: −0.95 to 4.18; *n* = 3), while running performance increased by 9.8% (95% CI: −3.28 to 16.78; *n* = 3). Only one study applied PNF stretching and reported an improvement in RE of 3.1% (see Table 3).

**TABLE 3:** Average changes in running economy and running performance parameters as related to stretching technique.

	**Running economy parameters**	**Running performance parameters**
Static stretching	+0.4% (*n* = 6)	−1.6% (*n* = 5)
Dynamic stretching	−0.8% (*n* = 3)	+9.8% (*n* = 3)
Proprioceptive neuromuscular facilitation stretching	+3.1% (*n* = 1)	n.a.

## Discussion

The literature review identified 11 studies that have investigated the effects of a single bout of stretching on RE and/or running performance. Bringing all the results together, there are conflicting reports about the effects of acute stretching prior to a running event. Some studies have reported that stretching has a negative effect on performance parameters (Wilson et al., 2010; Lowery et al., 2014) and RE (Wilson et al., 2010; Zourdos et al., 2012). This negative effect might be associated with a more pronounced ground contact time (Lowery et al., 2014). However, most of the studies have reported no changes in running performance (Allison et al., 2008; Mojock et al., 2011; Zourdos et al., 2012; Damasceno et al., 2014; Takizawa et al., 2015), RE (Hayes and Walker, 2007; Allison et al., 2008; Mojock et al., 2011; Damasceno et al., 2014; Takizawa et al., 2015; Yamaguchi et al., 2015), and heart rate response (Allison et al., 2008; Mojock et al., 2011), independent of the stretching technique used (static, dynamic). Nevertheless, positive effects have also been found. Godges et al. (1989) reported that static stretching (and partly also PNF stretching) had a positive effect on RE. Another research group (Yamaguchi et al., 2015, 2020) showed increased running performance following a dynamic stretching regimen, but without changes in RE.

To provide a clearer picture of the effects of a single bout of stretching on RE and running performance, the individual studies are first discussed in detail. The effects of the stretching duration and the different stretching techniques are then summarized.

The impact of six lower body static stretching exercises of 3 × 30 s on a 1-mile uphill treadmill run in 10 male distance runners was investigated by Lowery et al. (2014). Compared to the non-stretching condition, the static stretching induced a reduced 1-mile uphill run performance. A possible biomechanical explanation for the reduced running performance was found in a more pronounced ground contact time. The authors concluded that the higher ground contact time caused a “decrease in the efficiency to transfer of previously stored energy” (i.e., an adverse stretch-shortening cycle) and therefore a decrease in running performance. Similar results were reported by another research group with a similar static stretching protocol (six lower body exercises for 4 × 30 s) in male distance runners (Wilson et al., 2010), where the authors reported a decreased 30-min running performance and an impairment in RE (increased energy expenditure) in the stretching condition compared to the non-stretching control condition. Mojock et al. (2011) repeated the experiment with female long-distance runners, but with a slightly adapted stretching protocol (five lower body exercises for 2 × 60 s), and they reported no difference between the static stretching and non-stretching conditions in RE, calorie expenditure, heart rate, and endurance performance. The authors speculated that the discrepancy between the results in male (Wilson et al., 2010) and female (Mojock et al., 2011) distance runners is probably due to the less stiff muscle–tendon units in females, which might lead to less change in the muscle–tendon unit compliance following static stretching exercises. All in all, the study of Mojock et al. (2011) was the only study found in this scoping review which investigated the effects of stretching on RE and running performance in female participants. In total, we extracted the results for 148 participants, including 136 male participants but only 12 female participants. Since differences between females and males in the mechanical properties of the muscle–tendon unit (Konrad et al., 2017) and in the effects of stretching on running parameters (Wilson et al., 2010; Mojock et al., 2011) have been reported, we recommend that more studies be conducted on this topic with a female population. Furthermore, other studies including only male subjects have also reported no difference between static stretching and non-stretching conditions on running performance or economy as follows. The male runners in the study of Allison et al. (2008) performed eight (unilateral) static stretching exercises for 4 × 40 s. The authors reported a decrease in countermovement jump height and isometric strength, but no difference in RE (i.e., oxygen uptake, minute ventilation, energy expenditure, respiratory exchange ratio) or heart rate response, compared to the non-stretching condition. Moreover, the explored biomechanical variables (stride length, stride frequency) which could have explained possible physiological changes did not change. A further study reported no differences in a 3-km all-out run and a RE test of 11 male long-distance runners who either stretched statically for 3 × 30 s (seven exercises) or did not perform any stretching (Damasceno et al., 2014). However, similar to Allison et al. (2008), they found a detrimental effect on jump performance (drop jump height). Interestingly, subjects in the stretching condition performed the first 100 m of the 3-km run slower than those in the non-stretching condition. The authors assumed that the static stretching caused an impairment of the neuromuscular function, which resulted in a slower starting performance. Although Allison et al. (2008) and Damasceno et al. (2014) reported a decrease in muscle performance parameters, which could have been expected with such a stretching duration (Behm et al., 2016). This had no effect on running performance or metabolic parameters. Moreover, post-stretching dynamic activities likely minimize the negative effects of stretching (Behm et al., 2016). It can be assumed that the first 100 m in the 3-km performance run (Damasceno et al., 2014) can be compared to post-stretching dynamic activities, and hence the overall 3-km performance was not influenced by the stretching exercise. A shorter static stretching duration (1 × 20 s) of five muscle groups of the lower body was applied in the study of Takizawa et al. (2015), who reported no significant differences compared to the control group in the time to exhaustion or in RE (oxygen uptake).

Hayes and Walker (2007) reported that different stretching techniques [static stretching, progressive static stretching (similar to static stretching, with increasing load in the last 10 s of the stretch), dynamic stretching] had no impact on RE or steady-state oxygen uptake compared to the non-stretching controls. The seven male subjects had to perform five stretching exercises of 2 × 30 s each. Moreover, Godges et al. (1989) applied more intensive stretching protocols (static and PNF), including two muscles (hip flexors and hip extensors) and a stretching duration of 10 min/muscle, in their study of seven male subjects (non-runners) with limited hip flexion and/or extension flexibility. They reported that the static stretching induced an increase in RE in all three conditions (40, 60, and 80% of VO_2_ max), compared to the baseline measurement. With regard to PNF stretching, they found an increase in RE at 60% of VO_2_ max only. However, since Godges et al. (1989) tested subjects with limited hip flexibility, these results have to be interpreted with caution and should not be generalized. Saunders et al. (2004) suggested in their review that less flexible runners should aim for an optimum amount of flexibility which allows a more economical run. Thus, it can be assumed that, in the study of Godges et al. (1989), the less flexible participants achieved more beneficial flexibility (close to the optimum) for a running exercise due to the stretching exercise and therefore improved RE. It is therefore likely that, if the experiment of Godges et al. (1989) with the massive stretching duration of 600 s was repeated with normal flexible participants, the strength parameters would likely decrease dramatically (Behm et al., 2016), with negative effects on both RE and running performance. Yamaguchi et al. (2015, 2020) applied a dynamic stretching-like intervention (a type of gymnastics), including five exercises performed 10 times, as fast as possible, on running performance in male middle- or long-distance runners. They reported no changes in RE; however, the time to exhaustion and running distance were prolonged in the dynamic stretching group compared to those in the non-stretching control. No differences in running performance after a similar dynamic stretching regimen (10 exercises, 2 × 4 repetitions) were reported in 14 male runners by Zourdos et al. (2012). However, the authors found an impairment in RE (higher calorie expenditure) after the dynamic stretching intervention compared to the non-stretching condition.

### Stretching Duration

With regard to static stretching, except for the study of Hayes and Walker (2007; 60 s each muscle) and Takizawa et al. (2015; 20 s each muscle), the stretching duration of most of the included studies in this review varied from 90 s to 10 min for one muscle–tendon unit. Such a long stretching duration likely has a detrimental effect on muscle performance output. Two reviews (Behm et al., 2016; Chaabene et al., 2019) reported a greater loss in performance with static stretches of ≥60 s (−4.0 to −7.5%) compared to static stretches of <60 s (−1.0 to −2.0%). However, with regard to PNF stretching, only three out of 19 strength-based measures in the selected studies showed significant reductions after a single bout of stretching ranging from 28 s to 10 min (Behm et al., 2016). Kay and Blazevich (2012) pointed out in their review that, in three-quarters of the involved studies, a static stretch of less than 45 s did not affect muscle strength in terms of measured peak torque. More recently, our group showed that static stretching for 60 s increased the RoM of a joint, without changes in maximum isometric torque values or changes in the mechanical properties of the muscle–tendon unit (Konrad and Tilp, 2020). In a previous experiment, we also showed that dynamic muscle strength and the mechanical properties of the muscle–tendon unit (stiffness) did not change following 15 or 60 s of static stretching of multiple leg muscles (Stafilidis and Tilp, 2015). This is in accordance with the study of Nakamura et al. (2013), who did not observe any changes in passive resistive torque or muscle–tendon junction displacement (measured with B-mode ultrasound) after 1 min of stretching. In contrast, static stretching for longer than 60 s increases the RoM of a joint and decreases muscle stiffness (Kay et al., 2015; Konrad and Tilp, 2020), which might cause negative changes in muscle performance output (Kay and Blazevich, 2012). However, Allison et al. (2008) and also Damasceno et al. (2014) reported detrimental effects on strength and jump performance, but no changes in RE or related running performance parameters following a 90-s (Damasceno et al., 2014) or 160-s (Allison et al., 2008) static stretching exercise. Thus, it can be assumed that a decrease in strength or jump performance does not necessarily result in a decrease in running performance or RE. The reduction in muscle stiffness reported following a single stretching session (Konrad et al., 2017), which likely induces a decrease in elastic energy storage of the muscle–tendon unit (Asmussen and Bonde-Petersen, 1974), might have a higher impact on strength and jumping tasks compared to endurance running due to the higher speed required in these movements.

Furthermore, the average values of the included studies (about static stretching) showed more pronounced impairments in RE or running performance in studies that considered stretches of ≥120 s [performance: −1.8% (*n* = 2); RE: −0.03% (*n* = 4)] compared with studies that considered stretches of ≤90 s [performance: −1.4% (*n* = 3); RE: +1.0% (*n* = 3)]. These results indicate that running performance is impaired by medium (≤90 s: −1.4%) to long (≥120 s: −1.8%) stretching durations, while RE can be improved by medium stretching durations (≤90 s: +1.0%) but is either not affected or somewhat negatively affected by long stretching durations (≥120 s: −0.03%). A reason for the different effects of stretching on performance and economy could be that RE in the included studies was tested at submaximal levels, while the testing of running performance requires maximum effort. Sasaki and Neptune (2006) showed in their simulation study that the relative positive work of the serial elastic element in relation to the muscle fiber work is significantly different during walking (40%) and running (72%). Similarly, Hof et al. (2002) showed in their experiments that a different amount of work is performed by the serial elastic element at different walking and running speeds. This has substantial effects on RE as no metabolic energy is utilized for the release of elastic energy of the serial elastic component of the muscle–tendon unit. At one hand, a decrease in muscle–tendon stiffness due to stretching might therefore be beneficial for RE, which was determined at submaximal levels in the reviewed studies. On the other hand, the same decrease in muscle–tendon unit stiffness induced by long stretching durations might have decreased the direct force transmission during short and powerful movements and therefore decreased running performance. Thus, it would be interesting for future studies to test if the running performance in longer-lasting efforts (e.g., a marathon run) would also show a negative effect following stretching exercises.

However, this interpretation is also based on data from the less flexible non-athlete participants investigated by Godges et al. (1989). By excluding the results of this population (Godges et al., 1989), the average impairment of RE changes from −0.03 to −1.7% at stretching durations of ≥120 s. Thus, it is likely that normally flexible athletes will find a negative impact on RE if they stretch statically and for more than 120 s.

### Stretching Method

Static stretching led to a marginal average improvement in RE (0.4%) and a decrease in running performance (−1.6%) (see also Table 3). However, by again excluding the less flexible athletes and non-athletes tested by Godges et al. (1989), the average RE change shifts from a marginal improvement to a marginal impairment (−0.5%). By classifying the studies again according to the durations of 20–90 and ≥120 s in normally flexible athletes, the studies with the less pronounced stretching regimen (20–90 s) showed a small improvement in RE (1.0%) and an impairment (−1.7%) compared to the longer stretching regimen. However, the difference between stretching durations is not as clear for running performance (20–90 s: −1.4%; ≥120 s: −1.8%). Bringing the existing evidence about static stretching together, it is not recommended that healthy athletes with normal flexibility apply static stretches for 60 s or more prior to a running event when the focus is on increasing RE or running performance. However, it appears that less flexible runners can benefit from static stretching since Godges et al. (1989) reported a positive effect on RE following a 10-min static stretching exercise.

With regard to dynamic stretching, the included studies showed, on average, an impairment in RE (−0.8%) but an improvement in running performance of 9.8%. However, the result for running performance is based on two studies that reported improvements of 15.9 and 16.8% (Yamaguchi et al., 2015, 2020) and one study that reported significant impairments of 3.3% (Zourdos et al., 2012). The differences seem to be based on the stretching duration. Although it is difficult to determine the exact stretching durations for each muscle because dynamic stretching includes whole-body movements, in the studies of Yamaguchi et al. (2015, 2020), a total stretching duration of 217–220 s was applied, while longer total stretching durations were reported by Zourdos et al. (2012; 900 s).

Thus, the application of short-duration dynamic stretching is recommended, but not static stretching, when the goal is to increase running performance. If the goal is to maximize RE, a single stretching exercise for 60 s or longer should be avoided before running. There is only one study published so far that investigated PNF stretching in a non-athlete, less flexible population (Godges et al., 1989). Thus, we recommend that future studies should investigate the effect of PNF stretching exercises on RE and running performance to fill this gap in the literature.

### Post-stretching Dynamic Activities

Post-stretching dynamic activities following stretching exercise might be a possible approach to decrease the likelihood of a drop in performance following stretching exercise. Samson et al. (2012) compared the effects of general and general plus specific warm-ups with normal or dynamic stretching on springiness exercises (i.e., countermovement jump height or 20-m sprint time). All the stretching regimes were performed for 3 × 30 s for each muscle, resulting in a total stretching time of 90 s (per muscle). When a sport-specific warm-up was included (post-stretching), the 20-m sprint time following static and dynamic stretching showed an improvement compared to the static and dynamic stretching groups without a specific warm-up. Moreover, subjects that performed either a 5-s static stretch, a 30-s static stretch, or a five-repetition dynamic stretch for each muscle, including both a low-intensity (pre-stretching) and a high-intensity (post-stretching) warm-up, showed no deficit in springiness tasks (Blazevich et al., 2018). Moreover, Reid et al. (2018) reported increased vertical jump performance following 30 or 60 s of static stretching and no change of force produced at 100 ms when stretching was combined with a post-stretching comprehensive warm-up. In contrast, subjects that performed static stretches for 120 s (with the same comprehensive warm-up) showed no change in vertical jump performance or force produced at 100 ms. Bringing these findings together, there is evidence that post-stretching dynamic activities performed after static and dynamic stretching of up to 90 s increase springiness performance, while a longer stretching period (120 s) produces either a negative effect or has no effect. Since it has been shown that post-stretching dynamic activities are able to counteract a detrimental effect in performance, several authors (see Behm et al., 2016 for a review) have suggested including post-stretching dynamic activities in the warm-up regimes of athletes. This might also be a way for endurance athletes to increase their running performance or RE. Allison et al. (2008) and also Damasceno et al. (2014) reported detrimental effects in strength and jumping performance but no changes in RE or related running performance parameters following a single static stretching exercise. One could speculate that the post-stretching dynamic activities might have prevented the observed decreases in jumping and strength performance. Thus, this might have led to an increase in running performance and/or RE. However, to date, no study has investigated the effects of a single bout of stretching combined with post-stretching dynamic activities, which is a study design we recommend for future research.

### Stretching of Multiple Muscle Groups

The participants of almost every study included in this review of stretching and running performance/economy stretched several muscle groups prior to the running tests. However, it is known that more compliant muscle–tendon units of the knee extensors (Arampatzis et al., 2006), but stiffer plantar flexors (Gleim et al., 1990; Arampatzis et al., 2006; Hunter et al., 2011) or hamstring muscles (Gleim et al., 1990; Jones, 2002; Trehearn and Buresh, 2009), are advantageous for running performance/economy. On the one hand, stretching the plantar flexors or hamstrings will decrease muscle–tendon unit stiffness (e.g., when applied for >60 s) and eventually have a detrimental effect on running performance/economy, based on changes in the stretch shortening cycle (i.e., higher ground contact time; Lowery et al., 2014). On the other hand, a PNF stretching of the quadriceps muscles prior to running, with the goal to decrease tendon stiffness (Kay et al., 2015), might help to increase running performance/economy. This was already addressed by Allison et al. (2008), who speculated that a possible negative effect of hamstring and plantar flexor stretching might have canceled out the positive effect of quadriceps muscles stretching. This could explain the lack of overall effect of stretching on RE in their study. As a consequence, the authors underlined that the effect of an isolated bout of quadriceps stretching prior to a running event might be beneficial and needs to be investigated further.

### Different Flexibility Levels

To the best of our knowledge, to date, no study has tested the effects of a single bout of stretching on running performance/economy on athletes with different flexibility levels. This appears to be interesting as Godges et al. (1989) showed that intense stretching (10 min) of the hip flexors and hip extensors in less flexible participants led to an increase in RE. Since this result was in contrast to the other studies including participants with normal flexibility, one could assume that the less flexible participants benefit from a bout of stretching prior to running, while “normal” and “loose” subjects might not. A study design including athletes with different flexibility levels would likely produce individual recommendations for athletes according to their individual flexibility levels.

## Conclusion

Having considered the findings in the literature on the acute effects of stretching on running performance and RE, we recommend that dynamic stretching is performed (for a short duration of up to 220 s in total; Yamaguchi et al., 2015, 2020), but not static stretching, if the goal is to increase running performance when stretching is performed without further warm-up. Although small improvements in RE have been reported following static stretching durations of up to 90 s, no beneficial effect can be seen in running performance. Even though rigorous static stretching likely has no beneficial effect on running performance, a 54% reduction in acute muscle injuries has been reported with stretching (Behm et al., 2016). Therefore, static stretching, especially if applied for short durations and in combination with additional warm-up exercises, still has overall positive effects.

However, considering the application of stretching in sports practice, further conditions have to be considered to give recommendations. First of all, post-stretching dynamic activities must be implemented to decrease the likelihood of performance deficits (Behm et al., 2016). Furthermore, it can be suggested that targeted stretching of only the muscle groups for which greater compliance is beneficial for RE should be applied (i.e., a stretch of the quadriceps muscles only; Arampatzis et al., 2006). Moreover, since most of the included studies in this review performed stretching durations of far more than the critical duration of 60 s (with regard to strength deficits; e.g., Behm et al., 2016), a more sports practice-oriented approach of stretching in terms of duration must be applied. In addition, it should be considered that less flexible runners should aim to reach an optimum level of flexibility which allows a more economical run (Godges et al., 1989; Saunders et al., 2004). Thus, less flexible runners, at least, should stretch frequently (Saunders et al., 2004) and also prior to a running event (Godges et al., 1989; Saunders et al., 2004). Since the amount of studies on this topic is still very limited, we recommend that further studies be conducted including participants with different flexibility levels so as to be able to detect different group responses to stretching on RE and running performance.

## Data Availability Statement

The original contributions presented in the study are included in the article/supplementary material, further inquiries can be directed to the corresponding author/s.

## Author Contributions

AK, RM, and MN collaborated in the literature review and producing the figures and tables. AK, MN, KS, and MT collaborated in writing the manuscript. All the authors contributed to the article and approved the submitted version.

## Conflict of Interest

The authors declare that the research was conducted in the absence of any commercial or financial relationships that could be construed as a potential conflict of interest.
